# The Sideclose Technique: A Novel Method for Achieving Hemostasis With an Indwelling Impella CP

**DOI:** 10.1016/j.jscai.2024.102141

**Published:** 2024-05-18

**Authors:** Ethan Korngold, Jason Wollmuth

**Affiliations:** Providence Heart Institute, Providence St. Vincent Medical Center, Portland, Oregon

**Keywords:** complication, hemostasis, Impella

Access site bleeding remains a persistent challenge in patients with Impella CP catheters (Abiomed).^1^^,^^2^ Strategies for management include sheath manipulation, manual pressure, mattress suture placement, or preprocedural placement of Perclose suture-mediated closure devices (Abbott Vascular).^3^^,^^4^ Despite these maneuvers, bleeding around the repositioning sheath can occur, often leading to premature device removal. In this report, we describe the “sideclose” technique, a novel strategy to achieve hemostasis with the placement of an additional Perclose while maintaining an Impella CP uninterrupted.

The sideclose is a 2-person technique that begins with an Impella CP in place, the 14F peel-away sheath removed and the 9F to 13F tapered repositioning sheath within the vessel (Figure 1A). First, the sideport of the repositioning sheath is wired with a 0.035-inch J-wire (Figure 1B). While holding pressure at the arteriotomy site, the repositioning sheath is withdrawn over the Impella shaft while carefully maintaining the position of the Impella catheter. The backend of the 0.035-inch J-wire is pulled through the repositioning sheath (Figure 1C). A Perclose device is then placed over the 0.035-inch J-wire alongside the Impella shaft, in order to suture closed a portion of the arteriotomy (Figure 1D, E). Next, the Impella repositioning sheath is gently readvanced into the now smaller arteriotomy to achieve hemostasis (Figure 1F).

**Figure 1:**
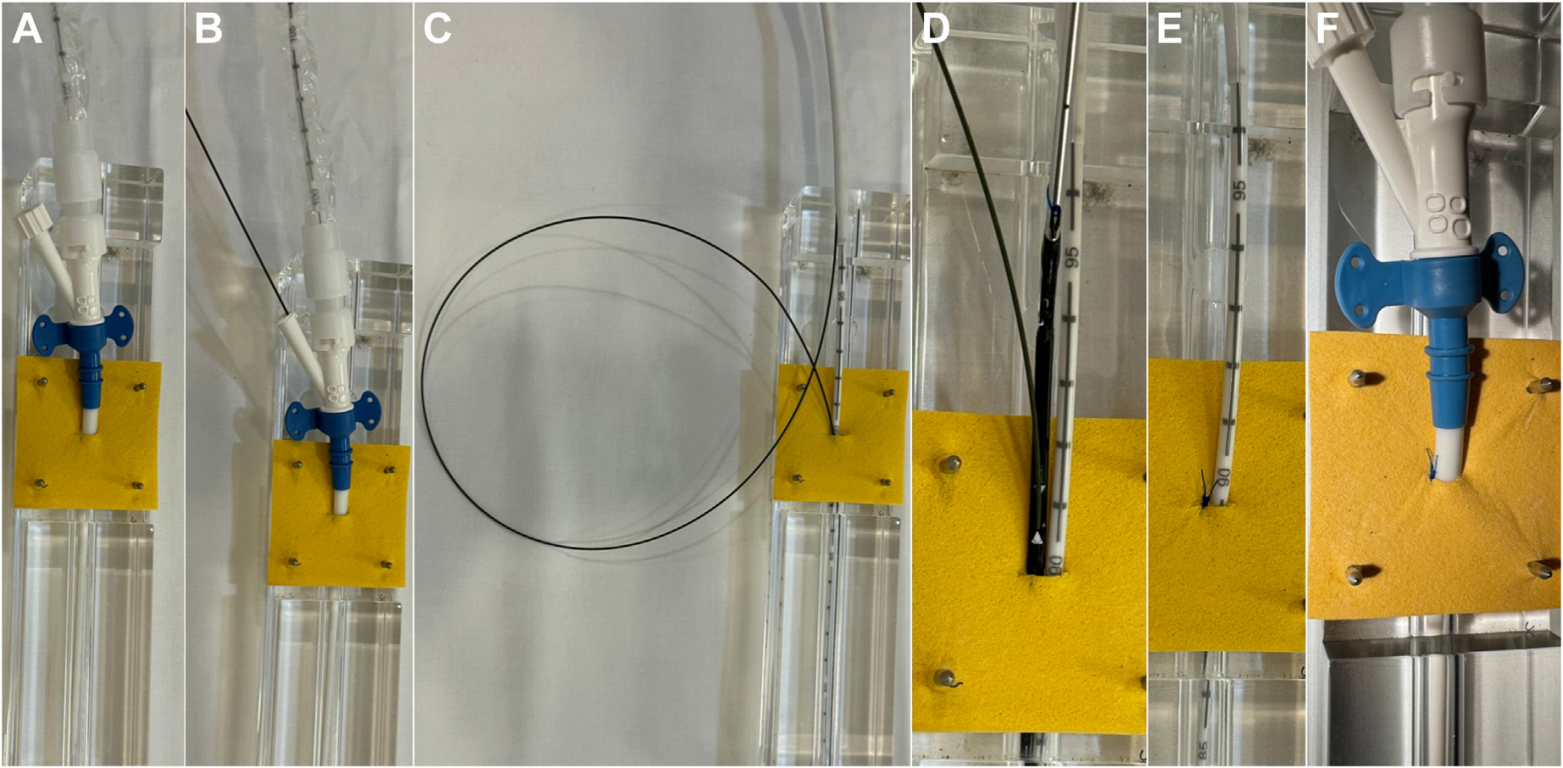
**Demonstration of the sideclose technique in a model.** (**A**) The Impella CP repositioning sheath within the arteriotomy. (**B**) The repositioning sheath side port is wired with a 0.035-inch J-wire. (**C**) The repositioning sheath is withdrawn while maintaining position of the Impella catheter, then the backend of the J-wire is pulled through the repositioning sheath. (**D**) A Perclose is placed alongside the Impella catheter. (**E**) The Perclose suture is tightened and trimmed. (**F**) The repositioning sheath is replaced within the partially sutured arteriotomy.

## Case report

A 65-year-old man with a history of end-stage renal disease, multivessel coronary artery disease, and an ejection fraction of 20% was admitted with decompensated heart failure and severe aortic stenosis. He underwent urgent transcatheter aortic valve replacement with a 26-mm SAPIEN 3 prosthesis via a 14F eSheath (Edwards Lifesciences). A single Perclose suture had been deployed prior to the introduction of the eSheath. Immediately after valve deployment, the patient became hypotensive and required cardiopulmonary resuscitation. Echocardiogram showed left ventricular standstill with no evidence of tamponade, coronary occlusion, or aortic dissection. The eSheath was removed and an Impella CP was placed. The patient stabilized within 20 minutes of the initiation of mechanical support but had persistent bleeding from the groin site, despite Impella sheath manipulation and tightening of the preclose suture. The sideclose technique was performed, resulting in immediate hemostasis. The patient stabilized in the intensive care unit; the Impella was removed the following day without complication by tightening the initially placed preclose suture. No additional closure or compression devices were required.

## Discussion

The sideclose technique can be performed immediately after Impella CP placement or at an interval, with or without the existence of preclose sutures, and does not require the interruption of mechanical circulatory support. During sideclose, care must be taken to maintain the position of the Impella catheter while the repositioning sheath is withdrawn and readvanced. Possible complications or concerns with the technique include inadvertent movement of the Impella catheter, iatrogenic femoral artery stenosis or occlusion due to the additional suture, femoral artery injury, or failure to achieve hemostasis. It is theoretically possible for the Perclose needles to puncture the Impella catheter and cause damage to the device; to avoid this, we recommend positioning the Perclose squarely alongside the Impella with needles aligned at the 12-o’clock position (Figure 1D). Heavily diseased, calcified, or narrow caliber femoral vessels would likely not be well suited to this technique. Further study of this technique is required to refine the method and establish its clinical utility. Along with meticulous vascular access techniques, preclose strategies and careful attention to sheath angle and positioning, the sideclose technique may be useful and definitive in managing bleeding at the Impella arteriotomy site.
